
JOURNAL INFORMATION
==============================
NLM Title Abbreviation: ScientificWorldJournal
Journal ID: ScientificWorldJournal
NLM Journal ID: 101131163
Journal ID: TSWJ

The Scientific World Journal

ISSN: 2356-6140
EISSN: 1537-744X
Publisher: Wiley

ARTICLE INFORMATION
==============================
PMCID: PMC5974746
PMID: 14755120
DOI: 10.1100/tsw.2003.120
Article ID: 937302
Article version: 1
Subjects: Editorial

Child Health and Human Development Domain After One Year

Merrick Joav *
Kandel Isack
Morad Mohammed
National Institute of Child Health and Human Development, Division for Mental Retardation, Ministry of Social Affairs, Jerusalem, Israel
*Joav Merrick: jmerrick@internet-zahav.net
Publication date: 2003
Electronic publication date: 2003 Dec 18
Volume: 3
First page: 1382
Last page: 1387
Copyright: Copyright © 2003 Joav Merrick et al.
Copyright year: 2003
License: This is an open access article distributed under the Creative Commons Attribution License, which permits unrestricted use, distribution, and reproduction in any medium, provided the original work is properly cited.
License URL: https://creativecommons.org/licenses/by/3.0/

Keywords: child health, human development, public health

==============================
Editorial
TheScientificWorldJOURNAL (2003) 3, 1382–1387
ISSN 1537-744X; DOI 10.1100/tsw.2003.120

### Child Health and Human Development Domain After One Year

Joav Merrick[1,*], Isack Kandel[2], and Mohammed Morad[3]

*[1]National Institute of Child Health and Human Development, Office of the Medical Director, Division for Mental Retardation, Ministry of Social Affairs, Box 1260, IL-91012 Jerusalem and Zusman Child Development Center, Division of Pediatrics and Community Health, Ben Gurion University, Beer-Sheva; [2]Faculty of Social Science, Department of Behavioral Sciences, Academic College of Judea and Samaria, Ariel; and [3]Division of Community Health, Ben Gurion University, Beer-Sheva, Israel*

**E-mail**: jmerrick@internet-zahav.net

*Received December 2, 2003; Accepted December 4, 2003; Published December 18, 2003*

---

**KEYWORDS:** child health, human development, public health

**DOMAINS:** child health and human development, medical care, nursing

---

### INTRODUCTION

One year with the Child Health and Human Development domain as part of TheScientificWorldJOURNAL is coming to a close and time has come to take stock of the year passed by.

It has been a very active year with many activities in the field of child health and human development. This has also been the European Year of People with Disabilities and many activities have been held in Europe in order to focus on this part of the population. The first author has just arrived home after a short visit to Rotterdam, Holland to participate in an historic event — the graduation of ten physicians who became the first specialists in intellectual disability medicine in the world after 3 years of study under the guidance of Professor Heleen Evenhuis, M.D., who in the year 2000, became the first professor of intellectual disability at the Erasmus University Medical Center in Rotterdam, Department of Family Medicine. In connection with the graduation on the 27th November of 2003, at the conclusion of the "European Year for People with Disabilities", an invitational conference of professionals from 12 different countries was held in Rotterdam concerning health care for individuals with intellectual disabilities. The meeting was organized by NVAVG (the Dutch Society of Physicians for Persons with Intellectual Disabilities), the MAMH (European Association of Intellectual Disabilities Medicine), and the Erasmus Medical Center Department of specialist training for physicians for people with intellectual disabilities, The aim was to finalize and accept a European Manifesto about "health care for people with intellectual disabilities"[1].

*Corresponding author.
©2003 with author.

### PEOPLE WITH INTELLECTUAL DISABILITY

People with intellectual disabilities are citizens of their countries and have an equal right to be included in society, whatever their level of disability. People with intellectual disabilities have many gifts and abilities, but also have special needs for which they need a choice of services to support those needs. People with intellectual disabilities have the same Human Rights as other citizens. People with intellectual disabilities have the right to equal participation in society. They must participate in all decisions that concern their lives (Inclusion Europe).

In the manifesto[1] below, the participants of the November 2003 Rotterdam Meeting regarded the Standard Rules on the Equalization of Opportunities for Persons with Disabilities, as adopted by the United Nations General Assembly, 48th session, resolution 48/96, annex, of 20 December 1993, as the political and moral foundation for this special population. The Standard Rules have been developed on the basis of the experience gained during the United Nations Decade of Disabled Persons (1983–1992), taking into consideration the following documents:

- The International Bill of Human Rights, comprising the Universal Declaration of Human Rights
- The International Covenant on Economic, Social and Cultural Rights
- The International Covenant on Civil and Political Rights
- The Convention on the Rights of the Child
- The Convention on the Elimination of All Forms of Discrimination against Women
- The World Programme of Action concerning Disabled Persons
- World Health Organization, International Classification of Impairments, Disabilities, and Handicaps (Geneva, 1980) and the International Classification of Functioning, Disability and Health (ICF) (Geneva, 2001)

Informed consent is essential in the relationship between the health professional and his client. Therefore, information for the client and his family about diagnostic procedures and therapies should be in an easily understandable format. People with intellectual disabilities and their representatives should influence all decisions about health care at every level of the health care organization.

In this document or manifesto (below), the word "health" is defined by the World Health Organization: *"Health is a state of complete physical, mental and social well-being and not merely the absence of disease or infirmity."*

### MANIFESTO ABOUT THE BASIC STANDARDS OF HEALTH CARE FOR PEOPLE WITH INTELLECTUAL DISABILITIES

The following criteria should be universally recognized and accepted as basic standards of adequate health care for individuals with intellectual disabilities.

1. **Optimal availability and accessibility to mainstream health services with primary care physicians playing a central role. This means that people with intellectual disabilities will:**
    a. Use mainstream health services.
    b. Receive more time for consultations in the clinic or in home visits, when needed.
    c. Receive adequate support in communication, when needed.
    d. Receive a proactive approach to their health needs.

e. Have no extra financial, physical, or legislative barriers to use mainstream services.
   f. Be able to participate in screening programs, in the same way as anybody else.
   g. Be supported in achieving and maintaining a healthy lifestyle that will prevent illness and encourage positive health outcomes.
   h. Receive understandable information about health and health promotion (also available to family and caregivers).
   i. Receive health care with good cooperation and coordination between different professionals.
2. **Health professionals (especially physicians, psychiatrists, dentists, nurses, and allied professionals) in mainstream health services will have competencies in intellectual disabilities and therefore in some of the more specific health problems of people with intellectual disabilities. This will require that:**
   a. Health professionals have a responsibility to achieve competencies in the basic standards of health care for people with intellectual disabilities.
   b. These competencies include the awareness that not all the health problems of people with intellectual disability are caused by their disability.
   c. All training programs for health professionals pay attention to intellectual disabilities, including the most common etiology, some frequent syndromes, etiology-related health problems, communication, legal and ethical aspects.
   d. Training in attitude and communicational skills is as important as clinical skills and therefore is part of the training programs.
   e. Guidelines on specific health issues are available through Internet, CD-ROM, or otherwise.
   f. Health care professionals in mainstream services have easy access to and are able to get advice from specialist colleagues without extra financial, practical, or legislative barriers.
3. **Health professionals (physicians, psychiatrists, dentists, nurses, and allied professionals) who are specialized in the specific health needs of individuals with intellectual disabilities are available as a back-up to mainstream health services. These professionals can advise, treat specific medical problems, or take over (a part of) the medical care for people with intellectual disabilities. This will require that:**
   a. Training programs be available for health professionals who want to gain competencies in health issues of people with intellectual disabilities.
   b. These specialists create and maintain networks with specialized colleagues in and outside of their own profession, in order to improve their knowledge and skills. This can be achieved by personal contacts or by creating (virtual) centers of expertise.
   c. Research on health issues of people with intellectual disabilities is stimulated in cooperation with academic centers. Academic Chairs in Intellectual Disability Medicine should be created to initiate, stimulate, and coordinate research projects.
4. **Health care for individuals with intellectual disabilities often needs a multidisciplinary approach.**
   a. Specific health assessments and/or treatments need coordination between different health professionals (e.g., visual and hearing impairment, mental health care, care for people with multiple and complex disability, care for the elderly, rehabilitation care).
   b. Specialist training for nurses and other caregivers is stimulated. This includes learning how to support and care for people with intellectual disabilities who have, for instance, sensory impairments, autistic spectrum disorders, epilepsy, mental health problems, behavioral/forensic problems, physical and complex disabilities, swallowing and feeding problems, and age-related problems.

5. **Health care for people with intellectual disabilities needs a proactive approach.**
   a. Participation in national screening programs should be encouraged.
   b. Anticipating health investigations on visual and hearing impairments and other frequent health problems should be evidence based and routinely available.
   c. General and specific health monitoring programs are developed and implemented. In the development of Health Indicator Systems special attention is paid to people with intellectual disabilities.
   d. Responsibility for the development of anticipating investigation programs and for their implementation must be clarified (primary care physicians, Public Health Doctors, or specialized physicians).
   e. People with intellectual disabilities and their families have a right to etiological investigations.

### CHILD HEALTH AND HUMAN DEVELOPMENT JOURNAL

During the last year, this journal has had contributions from Denmark, the United States, Norway, New Zealand, Australia, Uganda, Cambodia, and Israel with more than 50 papers in the form of several editorials, book reviews, reviews, and research papers.

The fields covered have been very broad with papers on disability; health needs for persons with disability; toxicology; creative problem solving; antenatal care; long-term follow-up after pregnancy; many aspects of quality of life measurement, philosophy, theory, and research results; ADD; and aspects of holistic medicine.

The editors, editorial, and review board have been very busy and active with peer review, but without the help of many other reviewers, we would not have been able to complete this vast task.

### ACKNOWLEDGMENTS AND THANKS TO THE UNKNOWN REVIEWERS

The quality of **TheScientificWorldJOURNAL Child Health and Human Development domain** is due to the contributors and reviewers. Many of the constructive and helpful peer reviews are performed by members of the editorial and review board. However, other colleagues who contribute their knowledge, time, skills, and efforts usually remain anonymous. Thus, in appreciation for their work we would like to present this annual listing of *previously* unknown reviewers for the year 2003.

Jane Allen, B.Sc., Nottingham, U.K.

Katherine Bergwerk, M.D., Los Angeles, U.S.

Malcolm Campbell, Manchester, U.K.

Ida Dancyger, M.D., New York, U.S.

Yehuda Danon, M.D., Petaq Tikva, Israel

Gary Diamond, M.D., Kfar Saba, Israel

Martin Fisher, M.D., New York, U.S.

Saralee Glaser, Ph.D., Tel Hashomer, Israel

Zachi Grossman, M.D., Tel Aviv, Israel

Uri Givon, M.D., Tel Hashomer, Israel

Uri Kramer, M.D., Tel Aviv, Israel

Lorraine V. Klerman, Dr. P.H., Waltham, MA, U.S.

Meir Lotan, B.P.T., M.Sc., Kfar Saba, Israel

Michal Hemmo Lotem, M.D., Petaq Tikva, Israel

Anthony S. Luder, M.D., Safed, Israel

Darby McElderry, Kentucky, U.S.

Pierre-Andre Michaud, M.D., Lausanne, Switzerland

Noele Nelson, M.P.H., Beer-Sheva, Israel

Eli Sachar, M.D., Haifa, Israel

Mike Stawski, M.D., Tel Aviv, Israel

Joan-Carles Suris, M.D., M.P.H., Barcelona, Spain

Roger S. Tonkin, M.D.C.M., F.R.C.P.C., O.B.C., British Columbia, Canada

Richard P. Tritter, B.A., J.D., Bat Yam, Israel

Søren Ventegodt, M.D., Copenhagen, Denmark

Gil Zalsman, M.D., Petaq Tikva, Israel

### REFERENCES

1. Carpenter, S., Meijer, M., and Scholte, F. (2003) European Manifesto on Basic Standards of Health Care for People with Intellectual Disability. Erasmus Medical Center, Rotterdam.
2. A full list of all publications in 2003 can be supplied through contact with the first author.

**This article should be referenced as follows**:

Merrick, J., Kandel, I., and Morad, M. (2003) Child health and human development domain after one year. *TheScientificWorldJOURNAL* **3**, 1382–1387.

### BIOSKETCHES

**Joav Merrick, M.D., D.M.Sc.,** is Professor of Child Health and Human Development affiliated with the Zusman Child Development Center and Division of Pediatrics and Division of Community Health at the Ben Gurion University, Beer-Sheva, Israel and presently the Medical Director of the Division for Mental Retardation, Ministry of Social Affairs, Jerusalem and the Director of the National Institute of Child Health and Human Development. He has numerous publications in the field of child and human development, rehabilitation, intellectual disability, disability, health, welfare, abuse, advocacy and prevention. Dr. Merrick received the Peter Sabroe

Child Award for outstanding work on behalf of Danish Children in 1985 and the International LEGO-Prize (“The Children’s Nobel Prize”) for an extraordinary contribution towards improvement in child welfare and well being in 1987. E-mail: jmerrick@internet-zahav.net. Website: www.nichd-israel.com

**Isack Kandel**, **M.A., Ph.D.,** is Senior Lecturer at the Faculty of Social Sciences, Department of Behavioral Sciences, the Academic College of Judea and Samaria, Ariel. During the period 1985–93, he was Director of the Division for Mental Retardation, Ministry of Labour and Social Affairs, Jerusalem, Israel. E-mail: Kandeli@aquanet.co.il

**Mohammed Morad, M.D.**, is Specialist in Family Medicine, Lecturer in Family Medicine at the Division of Community Health, Ben Gurion University of the Negev and the Medical Director of a large area clinic in the city of Beer-Sheva. He has publications on Bedouin health, health aspects, spiritual health, and aging in persons with intellectual disability, and is a presenter on topics such as health policy and services for the disadvantaged at national and international conferences. E-mail: morad62@barak-online.net
